# 5-Chloro-2-methyl­sulfanyl-6-(naphtha­len-1-yl­oxy)-1*H*-benzimidazole methanol monosolvate

**DOI:** 10.1107/S1600536813033709

**Published:** 2013-12-21

**Authors:** Miguel Flores-Ramos, Rafael Castillo, Alicia Hernández-Campos, Marcos Flores-Alamo

**Affiliations:** a Facultad de Química, Departamento de Farmacia, UNAM, México DF, 04510, Mexico; b Facultad de Química, Universidad Nacional Autónoma de México, México DF, 04510, Mexico

## Abstract

In the title compound, C_18_H_13_ClN_2_OS·CH_3_OH, the dihedral angle between the benzimidazole group and the naphth­yloxy moiety [82.89 (5)°] very near to orthogonality. The H atom in the five-membered ring is disordered with equal occupancies at the two N atoms and the H atom of the methano­lic hy­droxy group is disordered with equal occupancies over two sites at the O atom. The methanol mol­ecule acts as a hydrogen-bond acceptor for the amino H atom and donates a hydrogen bond to the nonprotonated ring N atom. As a result, chains are formed running along the *a* axis.

## Related literature   

For related literature on compound alpha, see: Rivera *et al.* (2004 ▶); Vera-Montenegro *et al.* (2003 ▶); Fairweather (2009 ▶); McConville *et al.* (2010 ▶). For the synthesis of compound alpha, see: Hernández *et al.* (2002 ▶).
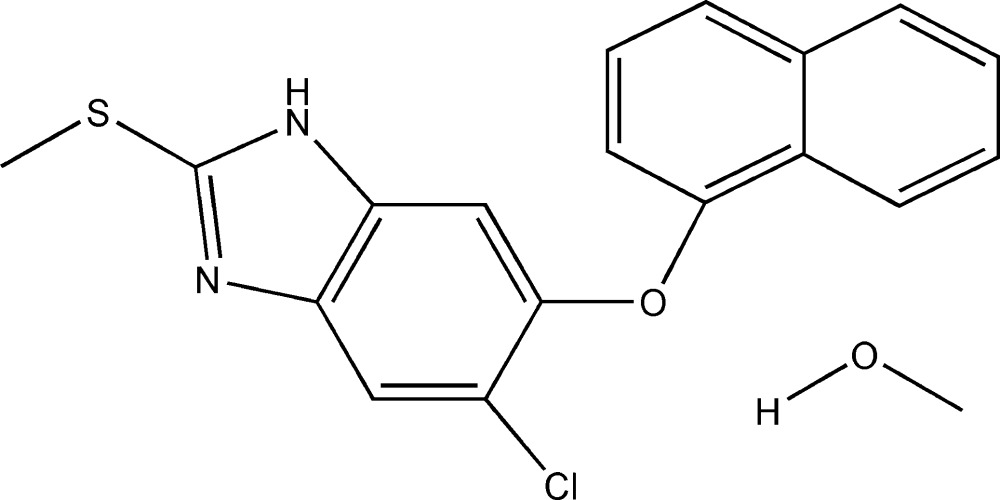


## Experimental   

### 

#### Crystal data  

C_18_H_13_ClN_2_OS·CH_4_O
*M* *_r_* = 372.85
Triclinic, 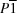

*a* = 7.4094 (6) Å
*b* = 9.1941 (8) Å
*c* = 14.4253 (11) Å
α = 73.978 (7)°
β = 75.315 (7)°
γ = 75.136 (7)°
*V* = 895.09 (14) Å^3^
*Z* = 2
Mo *K*α radiation
μ = 0.35 mm^−1^
*T* = 298 K
0.6 × 0.56 × 0.17 mm

#### Data collection  

Agilent Xcalibur (Atlas, Gemini) diffractometer
Absorption correction: analytical (*CrysAlis PRO*; Agilent, 2012 ▶) *T*_min_ = 0.785, *T*_max_ = 0.945
6945 measured reflections
4118 independent reflections
2706 reflections with *I* > 2σ(*I*)
*R*_int_ = 0.021

#### Refinement  

*R*[*F* ^2^ > 2σ(*F* ^2^)] = 0.049
*wR*(*F* ^2^) = 0.112
*S* = 1.03
4118 reflections
239 parameters
H atoms treated by a mixture of independent and constrained refinement
Δρ_max_ = 0.22 e Å^−3^
Δρ_min_ = −0.27 e Å^−3^

### 

Data collection: *CrysAlis PRO* (Agilent, 2012 ▶); cell refinement: *CrysAlis PRO*; data reduction: *CrysAlis RED* (Agilent, 2012 ▶); program(s) used to solve structure: *SHELXS97* (Sheldrick, 2008 ▶); program(s) used to refine structure: *SHELXL97* (Sheldrick, 2008 ▶); molecular graphics: *Mercury* (Macrae *et al.*, 2006 ▶); software used to prepare material for publication: *WinGX* (Farrugia, 2012 ▶).

## Supplementary Material

Crystal structure: contains datablock(s) global, I. DOI: 10.1107/S1600536813033709/bt6948sup1.cif

Structure factors: contains datablock(s) I. DOI: 10.1107/S1600536813033709/bt6948Isup2.hkl

Supporting information file. DOI: 10.1107/S1600536813033709/bt6948Isup3.cml

Additional supporting information:  crystallographic information; 3D view; checkCIF report

## Figures and Tables

**Table 1:** Hydrogen-bond geometry (Å, °)

*D*—H⋯*A*	*D*—H	H⋯*A*	*D*⋯*A*	*D*—H⋯*A*
O2—H1*O*⋯N2	0.94 (12)	1.87 (11)	2.768 (2)	158 (7)
O2—H2*O*⋯N1^i^	0.76 (11)	2.04 (11)	2.781 (3)	162 (9)
N1—H1*N*⋯O2^ii^	0.84 (8)	2.03 (8)	2.781 (3)	148 (7)
N2—H2*N*⋯O2	0.80 (9)	1.99 (9)	2.768 (2)	164 (7)

Symmetry codes: (i) 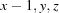; (ii) 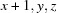.

**Fig. 1.**
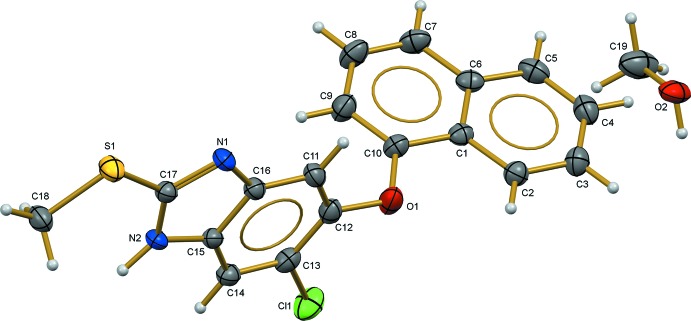
The molecular structure of the title compound. Displacement ellipsoids are drawn at the 40% probability level and H atoms are shown as circles of arbitrary size.

**Fig. 2.**
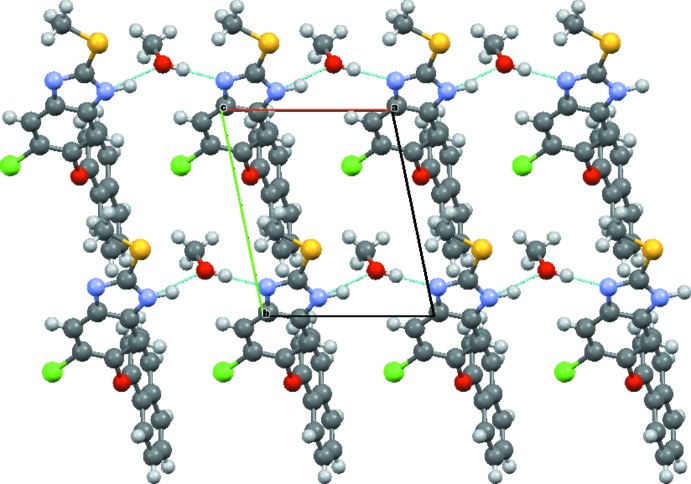
Crystal structure of the title compound viewed along the c-axis, showing the N—H···O and O—H···N hydrogen bond extending along the a-b plane. Only one site of the disordered H atoms is shown.

## supplementary crystallographic information

### 1. Comment

5-Chloro-2-(methylthio)-6-(1-naphthyloxy)-1*H*-benzimidazole (named compound Alpha) is a bioisostere of triclabendazole (TCBZ), the drug of choice for the treatment of fasciolosis caused by liver fluke in cattle and humans (Fairweather, 2009). Compound Alpha, synthesized by our research group, (Hernández *et al.*, 2002) proved to be as active as triclabendazole in cattle (Vera-Montenegro *et al.* 2003; Rivera *et al.*, 2004) and it acts in a similar way. Electron microscopy studies have shown that compound alpha affects the stability and integrity of microtubules in agreement with the action mechanisms of benzimidazoles anthelmintics (McConville *et al.*, 2010). However, the way in which TCBZ and compound Alpha interact at the molecular level with tubulin is still unknown. To establish the structure of compound alpha and its characteristics, we present the crystal structure of the title compound, useful for further modeling studies.

The asymmetric unit consist of the one molecule of 5-chloro-2-(methylthio)-6-(1-naphthyloxy)-1*H*-benzimidazole and one molecule of methanol (Figure 1). The benzimidazole group (plane 1) is coplanar with r.m.s. deviation of fitted atoms = 0.0592, in the same way for the naphthyloxy nucleus (plane 2) with r.m.s. deviation of fitted atoms = 0.0169, the angle between planes 1 and 2 are 83.99 (4) ° very near to orthogonality (90°), The C18 methylthio group is 0.1371 Å out of plane of the benzimidazole (plane 1).

The molecules are connected by N-H···O and O-H···N hydrogen bonds to chains running along the base vector [1 0 0] (Figure 2).

### 2. Experimental

The title compound, was prepared according to the procedure reported by Hernández *et al.* (2002). Single crystals were obtained from 2 g of compound alpha with 14 ml of methanol at 80 °C approximately, slow evaporation at room temperature of methanol afforded crystals suitable for X-ray diffraction resulting in 1.5 g of flat colourless crystals (yield 75%). 1*H*-NMR (DMSO-d6, 300 MHz) δ: 2.72 (3*H*, s, S—CH_3_), 6.67 (1*H*, d, J=7.2 Hz, H-2'), 7.31 (1*H*, s, H-4), 7.40 (1*H*, t, J1=7.9, J2=8.0 Hz, H-3'), 7.59–7.64 (2*H*, m, H-6', H-7'), 7.68 (1*H*, d, J=8.3 Hz, H-4'), 7.75 (1*H*, s, H-7), 7.97–8.03 (1*H*, m, H-5'), 8.28–8.32 (1*H*, m, H-8'), 12.79 (1*H*, br, NH) 13 C-NMR (DMSO-d6, 75 MHz) δ: 13.46 (S—CH_3_), 102.95 (C-7), 110.20 (C-2'), 113.16 (C-4), 119.70 (C-8'), 120.83 (C-5), 122.04 (C– 4'), 123.80 (C-8'a), 124.12 (C-3'), 124.66 (C-6'),125.25 (C-7'), 126.21 (C– 5'), 128.72 (C-4'a), 131.34 (C-3a), 133.08 (C-7a), 148.05 (C-1'), 150.75 (C-2), 152.03 (C-6). MS (EI) m/*z* 340 (*M*+), 305, 290.

### 3. Refinement

H atoms attached to C atoms were placed in geometrically idealized positions, and refined as riding on their parent atoms, with C—H distances fixed to 0.98 (methyl CH_3_), 0.99 (methylene CH_2_) and 1.00 Å (methine CH), and with *U*_iso_(H) = 1.5*U*_eq_(methyl C) or 1.2*U*_eq_(C). The H atoms bonded to N and O are disordered over two equally occupied positions.

### Crystal data

**Table:**

C_18_H_13_ClN_2_OS·CH_4_O	*Z* = 2
*M**_r_* = 372.85	*F*(000) = 388
Triclinic, *P*1	*D*_x_ = 1.383 Mg m^−^^3^
Hall symbol: -P 1	Mo *K*α radiation, λ = 0.71073 Å
*a* = 7.4094 (6) Å	Cell parameters from 1801 reflections
*b* = 9.1941 (8) Å	θ = 3.7–29.3°
*c* = 14.4253 (11) Å	µ = 0.35 mm^−^^1^
α = 73.978 (7)°	*T* = 298 K
β = 75.315 (7)°	Needle, colourless
γ = 75.136 (7)°	0.6 × 0.56 × 0.17 mm
*V* = 895.09 (14) Å^3^	

### Data collection

**Table:**

Agilent Xcalibur (Atlas, Gemini) diffractometer	4118 independent reflections
Graphite monochromator	2706 reflections with *I* > 2σ(*I*)
Detector resolution: 10.4685 pixels mm^-1^	*R*_int_ = 0.021
ω scans	θ_max_ = 29.4°, θ_min_ = 3.7°
Absorption correction: analytical (*CrysAlis PRO*; Agilent, 2012)	*h* = −9→9
*T*_min_ = 0.785, *T*_max_ = 0.945	*k* = −11→12
6945 measured reflections	*l* = −19→16

### Refinement

**Table:**

Refinement on *F*^2^	0 restraints
Least-squares matrix: full	H atoms treated by a mixture of independent and constrained refinement
*R*[*F*^2^ > 2σ(*F*^2^)] = 0.049	*w* = 1/[σ^2^(*F*_o_^2^) + (0.0371*P*)^2^ + 0.2903*P*] where *P* = (*F*_o_^2^ + 2*F*_c_^2^)/3
*wR*(*F*^2^) = 0.112	(Δ/σ)_max_ < 0.001
*S* = 1.03	Δρ_max_ = 0.22 e Å^−^^3^
4118 reflections	Δρ_min_ = −0.27 e Å^−^^3^
239 parameters	

### Special details

**Table:**

Geometry. All e.s.d.'s (except the e.s.d. in the dihedral angle between two l.s. planes) are estimated using the full covariance matrix. The cell e.s.d.'s are taken into account individually in the estimation of e.s.d.'s in distances, angles and torsion angles; correlations between e.s.d.'s in cell parameters are only used when they are defined by crystal symmetry. An approximate (isotropic) treatment of cell e.s.d.'s is used for estimating e.s.d.'s involving l.s. planes.
Refinement. H1N H1O and H2N H2O disordered over two sites with occupancies 0.50:0.50

### Fractional atomic coordinates and isotropic or equivalent isotropic displacement parameters (Å^2^)

**Table:**

	*x*	*y*	*z*	*U*_iso_*/*U*_eq_	Occ. (<1)
Cl1	0.69760 (10)	1.27046 (8)	0.30171 (5)	0.0685 (2)	
S1	1.35810 (8)	0.67687 (7)	−0.00019 (5)	0.05141 (19)	
O1	1.0904 (3)	1.32293 (17)	0.25994 (12)	0.0550 (4)	
N1	1.3436 (3)	0.9102 (2)	0.07959 (14)	0.0411 (5)	
N2	1.0553 (2)	0.8595 (2)	0.09667 (14)	0.0369 (4)	
C1	1.1882 (3)	1.4079 (2)	0.37612 (16)	0.0384 (5)	
C2	1.1589 (3)	1.5608 (3)	0.31968 (17)	0.0461 (6)	
H2	1.1211	1.5819	0.2598	0.055*	
C3	1.1857 (4)	1.6782 (3)	0.35235 (19)	0.0531 (6)	
H3	1.1658	1.7786	0.3145	0.064*	
C4	1.2426 (3)	1.6488 (3)	0.4420 (2)	0.0577 (7)	
H4	1.2598	1.7295	0.4638	0.069*	
C5	1.2729 (3)	1.5026 (3)	0.49713 (19)	0.0535 (6)	
H5	1.3111	1.4847	0.5566	0.064*	
C6	1.2482 (3)	1.3773 (3)	0.46688 (17)	0.0441 (5)	
C7	1.2791 (4)	1.2237 (3)	0.52271 (19)	0.0576 (7)	
H7	1.3215	1.2023	0.5814	0.069*	
C8	1.2483 (4)	1.1076 (3)	0.4923 (2)	0.0621 (7)	
H8	1.2699	1.0073	0.5302	0.075*	
C9	1.1837 (4)	1.1363 (3)	0.40414 (18)	0.0551 (6)	
H9	1.1605	1.0559	0.3843	0.066*	
C10	1.1556 (3)	1.2827 (3)	0.34797 (16)	0.0432 (5)	
C11	1.2346 (3)	1.1258 (3)	0.17067 (17)	0.0444 (5)	
H11	1.3536	1.1486	0.1626	0.053*	
C12	1.0750 (3)	1.2040 (2)	0.22222 (17)	0.0450 (6)	
C13	0.8955 (3)	1.1704 (2)	0.23444 (17)	0.0444 (5)	
C14	0.8714 (3)	1.0586 (2)	0.19396 (17)	0.0431 (5)	
H14	0.7518	1.0373	0.2013	0.052*	
C15	1.0327 (3)	0.9795 (2)	0.14192 (15)	0.0358 (5)	
C16	1.2126 (3)	1.0114 (2)	0.13086 (16)	0.0374 (5)	
C17	1.2424 (3)	0.8228 (2)	0.06164 (15)	0.0371 (5)	
C18	1.1676 (3)	0.5843 (3)	0.0082 (2)	0.0549 (6)	
H18A	1.2152	0.4998	−0.0242	0.082*	
H18B	1.0703	0.6574	−0.023	0.082*	
H18C	1.1152	0.5462	0.0761	0.082*	
O2	0.7136 (3)	0.7793 (2)	0.10784 (15)	0.0633 (6)	
C19	0.6753 (4)	0.6830 (4)	0.2003 (2)	0.0908 (11)	
H19A	0.6157	0.7443	0.2481	0.136*	
H19B	0.7922	0.6188	0.217	0.136*	
H19C	0.5916	0.6191	0.1996	0.136*	
H1O	0.837 (16)	0.801 (9)	0.088 (6)	0.136*	0.5
H2O	0.621 (15)	0.832 (10)	0.095 (7)	0.136*	0.5
H1N	1.463 (12)	0.896 (8)	0.068 (5)	0.109*	0.5
H2N	0.969 (12)	0.823 (8)	0.094 (5)	0.109*	0.5

### Atomic displacement parameters (Å^2^)

**Table:**

	*U*^11^	*U*^22^	*U*^33^	*U*^12^	*U*^13^	*U*^23^
Cl1	0.0649 (4)	0.0543 (4)	0.0730 (5)	0.0010 (3)	0.0026 (4)	−0.0200 (3)
S1	0.0344 (3)	0.0530 (4)	0.0709 (4)	−0.0078 (3)	−0.0067 (3)	−0.0249 (3)
O1	0.0821 (12)	0.0371 (9)	0.0522 (10)	−0.0096 (8)	−0.0300 (9)	−0.0073 (8)
N1	0.0305 (9)	0.0437 (11)	0.0514 (12)	−0.0136 (9)	−0.0082 (9)	−0.0091 (9)
N2	0.0271 (9)	0.0385 (10)	0.0459 (11)	−0.0081 (8)	−0.0107 (8)	−0.0064 (8)
C1	0.0327 (11)	0.0412 (12)	0.0372 (12)	−0.0052 (9)	−0.0042 (9)	−0.0067 (10)
C2	0.0475 (13)	0.0459 (13)	0.0432 (13)	−0.0124 (11)	−0.0097 (11)	−0.0040 (11)
C3	0.0573 (15)	0.0460 (14)	0.0558 (16)	−0.0179 (12)	−0.0074 (12)	−0.0077 (12)
C4	0.0544 (15)	0.0576 (17)	0.0684 (18)	−0.0211 (13)	−0.0071 (14)	−0.0217 (14)
C5	0.0445 (13)	0.0726 (18)	0.0486 (15)	−0.0121 (13)	−0.0108 (11)	−0.0202 (13)
C6	0.0348 (11)	0.0528 (14)	0.0418 (13)	−0.0048 (10)	−0.0065 (10)	−0.0105 (11)
C7	0.0615 (16)	0.0619 (17)	0.0460 (15)	−0.0019 (13)	−0.0220 (13)	−0.0057 (13)
C8	0.0781 (19)	0.0462 (15)	0.0522 (16)	−0.0023 (13)	−0.0206 (14)	0.0024 (12)
C9	0.0701 (17)	0.0399 (13)	0.0539 (16)	−0.0066 (12)	−0.0181 (13)	−0.0068 (12)
C10	0.0450 (13)	0.0396 (12)	0.0413 (13)	−0.0042 (10)	−0.0110 (10)	−0.0050 (10)
C11	0.0422 (12)	0.0447 (13)	0.0499 (14)	−0.0179 (11)	−0.0130 (11)	−0.0047 (11)
C12	0.0574 (15)	0.0364 (12)	0.0428 (13)	−0.0109 (11)	−0.0159 (11)	−0.0044 (10)
C13	0.0457 (13)	0.0371 (12)	0.0427 (13)	−0.0015 (10)	−0.0075 (10)	−0.0041 (10)
C14	0.0337 (11)	0.0414 (12)	0.0504 (14)	−0.0082 (10)	−0.0100 (10)	−0.0021 (11)
C15	0.0315 (10)	0.0339 (11)	0.0404 (12)	−0.0079 (9)	−0.0121 (9)	−0.0003 (9)
C16	0.0327 (11)	0.0373 (12)	0.0405 (12)	−0.0097 (9)	−0.0078 (9)	−0.0030 (10)
C17	0.0318 (11)	0.0378 (11)	0.0406 (12)	−0.0084 (9)	−0.0095 (9)	−0.0033 (10)
C18	0.0492 (14)	0.0552 (15)	0.0708 (17)	−0.0150 (12)	−0.0165 (13)	−0.0228 (13)
O2	0.0351 (9)	0.0765 (13)	0.0721 (13)	−0.0194 (9)	−0.0170 (9)	0.0068 (10)
C19	0.0643 (19)	0.110 (3)	0.082 (2)	−0.0294 (19)	−0.0245 (17)	0.025 (2)

### Geometric parameters (Å, º)

**Table:**

Cl1—C13	1.737 (2)	C7—C8	1.349 (4)
S1—C17	1.736 (2)	C7—H7	0.93
S1—C18	1.790 (2)	C8—C9	1.405 (3)
O1—C12	1.389 (3)	C8—H8	0.93
O1—C10	1.394 (3)	C9—C10	1.361 (3)
N1—C17	1.341 (3)	C9—H9	0.93
N1—C16	1.381 (3)	C11—C12	1.372 (3)
N1—H1N	0.84 (8)	C11—C16	1.390 (3)
N2—C17	1.338 (3)	C11—H11	0.93
N2—C15	1.386 (3)	C12—C13	1.399 (3)
N2—H2N	0.80 (9)	C13—C14	1.381 (3)
C1—C2	1.410 (3)	C14—C15	1.386 (3)
C1—C10	1.413 (3)	C14—H14	0.93
C1—C6	1.421 (3)	C15—C16	1.398 (3)
C2—C3	1.365 (3)	C18—H18A	0.96
C2—H2	0.93	C18—H18B	0.96
C3—C4	1.396 (3)	C18—H18C	0.96
C3—H3	0.93	O2—C19	1.388 (3)
C4—C5	1.354 (3)	O2—H1O	0.94 (12)
C4—H4	0.93	O2—H2O	0.76 (11)
C5—C6	1.407 (3)	C19—H19A	0.96
C5—H5	0.93	C19—H19B	0.96
C6—C7	1.412 (3)	C19—H19C	0.96
			
C17—S1—C18	101.75 (11)	O1—C10—C1	114.46 (18)
C12—O1—C10	117.61 (16)	C12—C11—C16	117.8 (2)
C17—N1—C16	105.61 (17)	C12—C11—H11	121.1
C17—N1—H1N	125 (5)	C16—C11—H11	121.1
C16—N1—H1N	129 (5)	C11—C12—O1	119.4 (2)
C17—N2—C15	105.35 (17)	C11—C12—C13	121.4 (2)
C17—N2—H2N	130 (5)	O1—C12—C13	119.2 (2)
C15—N2—H2N	124 (5)	C14—C13—C12	121.3 (2)
C2—C1—C10	123.0 (2)	C14—C13—Cl1	118.85 (18)
C2—C1—C6	119.1 (2)	C12—C13—Cl1	119.81 (18)
C10—C1—C6	117.9 (2)	C13—C14—C15	117.3 (2)
C3—C2—C1	120.5 (2)	C13—C14—H14	121.4
C3—C2—H2	119.8	C15—C14—H14	121.4
C1—C2—H2	119.8	N2—C15—C14	130.70 (19)
C2—C3—C4	120.6 (2)	N2—C15—C16	107.82 (18)
C2—C3—H3	119.7	C14—C15—C16	121.4 (2)
C4—C3—H3	119.7	N1—C16—C11	131.58 (19)
C5—C4—C3	120.0 (2)	N1—C16—C15	107.67 (18)
C5—C4—H4	120	C11—C16—C15	120.7 (2)
C3—C4—H4	120	N2—C17—N1	113.55 (19)
C4—C5—C6	121.8 (2)	N2—C17—S1	126.69 (16)
C4—C5—H5	119.1	N1—C17—S1	119.75 (15)
C6—C5—H5	119.1	S1—C18—H18A	109.5
C5—C6—C7	123.1 (2)	S1—C18—H18B	109.5
C5—C6—C1	118.0 (2)	H18A—C18—H18B	109.5
C7—C6—C1	118.9 (2)	S1—C18—H18C	109.5
C8—C7—C6	121.1 (2)	H18A—C18—H18C	109.5
C8—C7—H7	119.5	H18B—C18—H18C	109.5
C6—C7—H7	119.5	C19—O2—H1O	116 (5)
C7—C8—C9	120.8 (2)	C19—O2—H2O	110 (7)
C7—C8—H8	119.6	O2—C19—H19A	109.5
C9—C8—H8	119.6	O2—C19—H19B	109.5
C10—C9—C8	119.5 (2)	H19A—C19—H19B	109.5
C10—C9—H9	120.3	O2—C19—H19C	109.5
C8—C9—H9	120.3	H19A—C19—H19C	109.5
C9—C10—O1	123.7 (2)	H19B—C19—H19C	109.5
C9—C10—C1	121.8 (2)		
			
C10—C1—C2—C3	177.5 (2)	C10—O1—C12—C13	101.8 (2)
C6—C1—C2—C3	−0.8 (3)	C11—C12—C13—C14	−0.9 (3)
C1—C2—C3—C4	0.1 (4)	O1—C12—C13—C14	176.64 (19)
C2—C3—C4—C5	0.4 (4)	C11—C12—C13—Cl1	179.13 (17)
C3—C4—C5—C6	−0.1 (4)	O1—C12—C13—Cl1	−3.4 (3)
C4—C5—C6—C7	179.9 (2)	C12—C13—C14—C15	0.9 (3)
C4—C5—C6—C1	−0.5 (3)	Cl1—C13—C14—C15	−179.11 (16)
C2—C1—C6—C5	1.0 (3)	C17—N2—C15—C14	−177.1 (2)
C10—C1—C6—C5	−177.3 (2)	C17—N2—C15—C16	0.6 (2)
C2—C1—C6—C7	−179.4 (2)	C13—C14—C15—N2	177.4 (2)
C10—C1—C6—C7	2.3 (3)	C13—C14—C15—C16	0.0 (3)
C5—C6—C7—C8	178.0 (2)	C17—N1—C16—C11	178.3 (2)
C1—C6—C7—C8	−1.6 (4)	C17—N1—C16—C15	0.0 (2)
C6—C7—C8—C9	−0.2 (4)	C12—C11—C16—N1	−177.1 (2)
C7—C8—C9—C10	1.2 (4)	C12—C11—C16—C15	1.0 (3)
C8—C9—C10—O1	−179.4 (2)	N2—C15—C16—N1	−0.4 (2)
C8—C9—C10—C1	−0.5 (4)	C14—C15—C16—N1	177.52 (19)
C12—O1—C10—C9	−7.8 (3)	N2—C15—C16—C11	−178.92 (19)
C12—O1—C10—C1	173.2 (2)	C14—C15—C16—C11	−1.0 (3)
C2—C1—C10—C9	−179.5 (2)	C15—N2—C17—N1	−0.6 (2)
C6—C1—C10—C9	−1.3 (3)	C15—N2—C17—S1	178.42 (16)
C2—C1—C10—O1	−0.5 (3)	C16—N1—C17—N2	0.4 (2)
C6—C1—C10—O1	177.75 (18)	C16—N1—C17—S1	−178.71 (15)
C16—C11—C12—O1	−177.63 (18)	C18—S1—C17—N2	−5.5 (2)
C16—C11—C12—C13	−0.1 (3)	C18—S1—C17—N1	173.50 (18)
C10—O1—C12—C11	−80.6 (3)		

### Hydrogen-bond geometry (Å, º)

**Table:**

*D*—H···*A*	*D*—H	H···*A*	*D*···*A*	*D*—H···*A*
O2—H1*O*···N2	0.94 (12)	1.87 (11)	2.768 (2)	158 (7)
O2—H2*O*···N1^i^	0.76 (11)	2.04 (11)	2.781 (3)	162 (9)
N1—H1*N*···O2^ii^	0.84 (8)	2.03 (8)	2.781 (3)	148 (7)
N2—H2*N*···O2	0.80 (9)	1.99 (9)	2.768 (2)	164 (7)

Symmetry codes: (i) *x*−1, *y*, *z*; (ii) *x*+1, *y*, *z*.
